# Combined effects of body posture and three-dimensional wing shape enable efficient gliding in flying lizards

**DOI:** 10.1038/s41598-022-05739-1

**Published:** 2022-02-02

**Authors:** Pranav C. Khandelwal, Tyson L. Hedrick

**Affiliations:** 1 Max Planck Institute for Intelligent Systems, 70569 Stuttgart, Germany; 2 Department of Biology, University of North Carolina at Chapel Hill, Chapel Hill, NC 27599 USA

**Keywords:** Animal behaviour, Biomechanics

## Abstract

Gliding animals change their body shape and posture while producing and modulating aerodynamic forces during flight. However, the combined effect of these different factors on aerodynamic force production, and ultimately the animal’s gliding ability, remains uncertain. Here, we quantified the time-varying morphology and aerodynamics of complete, voluntary glides performed by a population of wild gliding lizards (*Draco dussumieri)* in a seven-camera motion capture arena constructed in their natural environment. Our findings, in conjunction with previous airfoil models, highlight how three-dimensional (3D) wing shape including camber, planform, and aspect ratio enables gliding flight and effective aerodynamic performance by the lizard up to and over an angle of attack (AoA) of 55° without catastrophic loss of lift. Furthermore, the lizards maintained a near maximal lift-to-drag ratio throughout their mid-glide by changing body pitch to control AoA, while simultaneously modulating airfoil camber to alter the magnitude of aerodynamic forces. This strategy allows an optimal aerodynamic configuration for horizontal transport while ensuring adaptability to real-world flight conditions and behavioral requirements. Overall, we empirically show that the aerodynamics of biological airfoils coupled with the animal’s ability to control posture and their 3D wing shape enable efficient gliding and adaptive flight control in the natural habitat.

## Introduction

**Figure 1:**
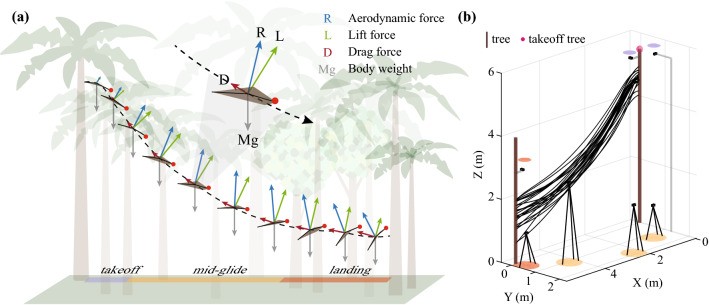
Illustration of the body posture and forces acting during a glide and the field motion capture arena. (**a**) Free body diagram showing the forces experienced by a gliding animal at various stages of the complete glide as well as the change in body orientation. The glide is further divided into the three distinct glide phases of takeoff, mid-glide, and landing. (**b**) A scaled illustration of the motion capture arena showing all 24 smoothed glides (see SI-3.2) and the seven camera positions used to collect 3D kinematic data. The seven cameras are divided into three groups (color coded) based on the part of the glide they record. The takeoff cameras are marked by purple discs, glide cameras with orange, and landing cameras with red.

The aerodynamics of gliding animals are deceptively simple. To move through air, animals modify their body to form an airfoil and use it to generate aerodynamic forces by trading their height above the surface (i.e., potential energy) for kinetic energy. The generated lift and drag forces counteract the pull of gravity during aerial descent, and change in magnitude and direction as the animal gains or loses speed and makes postural adjustments along with changes in their airfoil shape^1–10^ (Fig. 1a and movie S1). Together, these dynamic changes in the animal’s body posture and morphology, and consequently the aerodynamic force production, allows the animal to execute a variety of glide trajectories and achieve different tasks including mating, foraging, territoriality, and predatory avoidance, while accommodating their habitat’s spatial complexity^11^.

The underlying aerodynamic performance that enables these well-choreographed gliding behaviors emerges from the interaction between the animal’s compliant, low aspect ratio (AR, Table 1) airfoil and the surrounding air, an interaction which does not follow the characteristics typical of rigid, fixed-wing airfoils operating at higher Reynolds number^12^ (Re, Table 1). Aerodynamic performance is typically expressed as a drag polar plot showing the coefficient of lift (C_L_) versus the coefficient of drag (C_D_) through a range of angles of attack (AoA, Table 1). Positions on this plot reveal lift-drag combinations of interest such as those that produce the greatest glide distance, the maximum lift, or maximum total force, as well as the sensitivity of airfoil performance to variation in AoA. Although the airfoils of gliding animals possess fewer degrees of freedom than the flapping wings of birds and bats, characterizing the performance and control of their actuated, compliant, and low AR airfoils has proven surprisingly difficult. Numerical simulations and wind tunnel tests of static animal models have provided an estimation of a drag polar curve for gliding snakes, lizards, squirrels, and fish^1,13–17^, but do not address the aerodynamic effects of a compliant airfoil or mid-air postural changes. Kinematic studies of gliding squirrels and sugar gliders have shown the expected significant correlation between AoA and aerodynamic force coefficients, but methodological limitations forced these studies to examine a brief segment of the glide (< 0.38 s), lacking information needed to examine control of force production or effects of airfoil compliance throughout the entire glide^2,3,18^. Overall, despite prior work on a variety of gliding species, the combined effect of changes in body posture and airfoil properties on the animal’s aerodynamic performance and flight control, and ultimately, the animal’s gliding ability, remains unclear.

**Table 1:** Brief definitions of terminology.

Reynolds number (Re)	Dimensionless number used to categorize the inertial versus viscous properties of the fluid
Planform	The outline of the airfoil (shape) when projected on a horizontal plane
Chord	Distance between the leading and trailing edge of the wing
Aspect ratio (AR)	Ratio of the wingspan to the wing chord length
Angle of attack (AoA)	Angle made by the airfoil relative to the wind
Camber	The curvature of the wing in the chordwise direction
Dihedral angle	Upward angle made by the wing with respect to the horizontal plane

**Figure 2:**
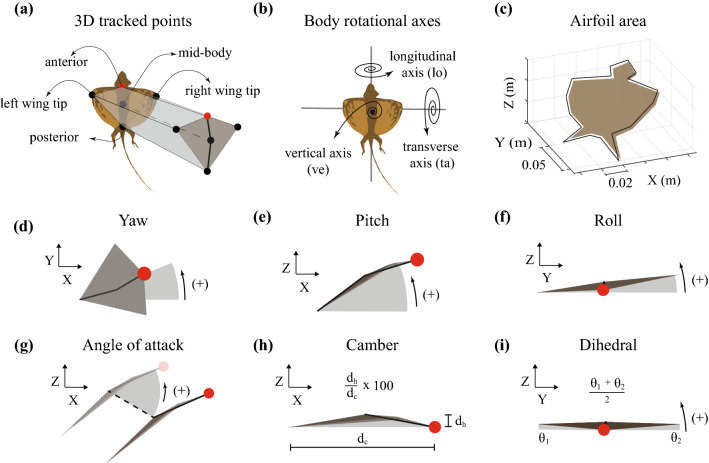
An illustration of the tracked body points and the metrics calculated for each glide. (**a**) Illustration showing the five body points tracked per frame for each glide. (**b**) The three axes about which the lizard can reorient in flight. Longitudinal axis (**lo**) about which the lizard rolls. Transverse axis (**ta**) about which the lizard changes pitch. Vertical axis (**ve**) about which the lizard changes yaw. (**c**) Illustration showing the airfoil area used to calculate the aerodynamic force coefficients. The area was calculated by fitting a plane to the 17 3D points tracked around the lizard in a single frame during the glide when the patagium was completely stretched open. (**d**) Yaw angle is the rotation about the (**ve**) from the X–Z plane, positive yaw is to the lizard’s left. (**e**) Pitch angle is the rotation about the (**ta**) from the X–Y plane, positive pitch raises the head upward. (**f**) Roll angle is the rotation about the (**lo**) from the Z–Y plane, positive roll raises the left-wing tip. (**g**) Angle of attack (AoA) is the angle made by the airfoil surface relative to the direction of motion (airflow), positive AoA is counterclockwise. (**h**) Camber is the convexity (concave down) of the airfoil from the leading to the trailing edge. The formula provided shows the calculation for % camber. (**i**) Dihedral angle is the average of the upward angle made by either side of the wing with the X–Y plane.

Here, we address these challenges by performing detailed aerodynamic measurements in a population of wild-caught gliding lizards (*Draco dussumieri*) executing complete, voluntary glides in a seven-camera motion capture arena constructed in their natural environment (Fig. 1b, also see Supplementary Information). We derived the three-dimensional (3D) kinematics from five body landmarks allowing measurement of body posture, airfoil orientation, and airfoil camber in addition to the overall glide trajectory (Fig. 2). Furthermore, we recorded the masses and the in-flight airfoil surface area of the individuals to calculate the instantaneous values of C_L_ and C_D_ for the complete glide (Fig. S1c and Fig. 2c). Using these metrics, we constructed a real-world aerodynamic performance landscape for the lizard and tested the following hypotheses. First, that the lizard’s aerodynamic performance would be representative of the performance of physical models of low AR compliant airfoils at comparable Re^19^. The lizard’s drag polar curve will have the highest lift-to-drag ratio (but smallest C_L_ and C_D_ values) occurring at low AoA followed by the airfoil transitioning to a “soft” stall with an increasing C_D_ and nearly constant C_L_ at higher AoA^19^. Second, as a consequence of the lizard’s compliant airfoil along with previous observations of in-flight camber in mammalian gliders and *Draco* gliding lizards^2,3,18,20,21^, we hypothesized that *Draco* gliding lizards should also vary camber during the glide, affecting their aerodynamic force production. A higher airfoil camber would lead to greater C_L_ and C_D_ as seen in compliant wing models^19,22^. Finally, since the primary *Draco* wing is formed by the activation of the intercostal musculature to rotate the ribs and stretch open the patagial membrane laterally, it restricts wing movement outside the dorsal plane of the body^23,24^. Similar anatomical restrictions are seen in other gliding species including mammalian gliders, gliding snakes, and invertebrate gliders^20,23,25–28^. Therefore, we hypothesized that the aerodynamic forces in *Draco* gliding lizards are primarily controlled by varying the body orientation (specifically body pitch) and consequently the AoA to enable a high lift-to-drag ratio during the mid-glide to cover horizontal distance, and high lift and drag forces at landing to minimize elevation loss while also reducing kinetic energy before touchdown.

## Results

A total of 24 complete glides from 7 males and 7 females were used for kinematic and aerodynamic analysis. Glides were recorded in a field motion capture arena consisting of a single fixed takeoff and landing tree spaced 5.50 m apart with no obstacles in between (Fig. 1a; also see Fig. S2). The average glide duration was 1.69 ± 0.05 s (mean ± sd, n = 14) with lizards reaching a maximum speed of 5.04 ± 0.26 ms^−1^. The average overall glide angle was − 28.3 ± 2.5° and corresponded to a theoretical equilibrium glide with a lift-to-drag ratio of ~ 2.0. However, none of the glides reached equilibrium, all showed continuous changes in acceleration and speed in the X, Y, and Z directions throughout the glide. The yaw and roll angles were highly variable and close to 0° across all glides; the average yaw was − 3.7 ± 3.6° and the average roll was − 0.4 ± 4.5°.

### Variation in pitch and airfoil properties during the glide

**Figure 3:**
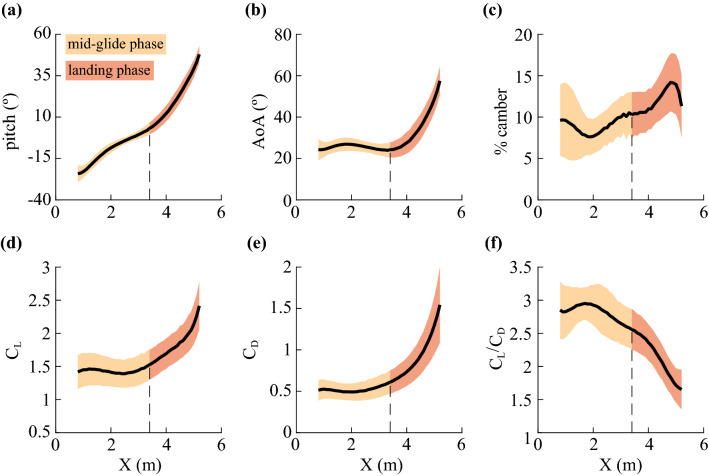
Average glide mechanics across all 14 individuals in the field motion capture arena. The solid black line shows the average value, and the shaded area shows ± 1 standard deviation. The vertical dashed line denotes the start of the landing phase calculated as the average of start of landing phase for all glides. Overall, the analysis covers ~ 82% of the complete glide. Panels (**a**–**c**) show the overall variation in pitch, AoA, and camber. Note the steady increase in pitch in the mid-glide phase and a mostly constant AoA of approximately 25° with varying percentage camber. Panels (**d**–**f**) show the change in average force coefficients and their ratio with glide progression. Note the steep drop in lift-to-drag ratio during the landing phase.

During takeoff, lizards exhibited a high degree of variation in body pitch. However, after entering the mid-glide phase, body pitch stereotypically increased at an average rate of 11.1 ± 2.7° m^−1^ (mean ± sd, n = 14) closely matching the trajectory shallowing rate during mid-glide of 11.0 ± 1.5° m^−1^. The landing maneuver saw the average body pitch rate increased to 25.9 ± 5.0° m^−1^ (Fig. 3a). Finally, at the end of the landing phase, the body pitch reached an average value of 51.2 ± 5.0° with the lizard rotating its head dorsally and extending the forelimbs forward for touchdown.

During mid-glide, the instantaneous changes in body orientation and glide angle combined to hold airfoil AoA constant at 25.3 ± 2.8° (Fig. 3b). Also at this time, the lizard’s airfoil maintained a concave downwards profile with an average percentage camber of 8.97 ± 1.97 (Fig. 3c) and a dihedral angle of 7.9 ± 2.1°, i.e., the camber height was ~ 9% of the chord length and the wing tips were elevated at ~ 8° compared to the base of the leading edge of the airfoil. After entering the landing phase, the AoA rapidly increased at a rate of 19.2 ± 4.6° m^−1^, reaching 60.0 ± 7.0° just before touchdown. The landing phase also saw lizards using a significantly higher average percentage camber of 11.97 ± 2.43 (Two-sample t-test, p = 0.001) compared to the mid-glide phase but with a similar dihedral angle of 8.2 ± 2.5°.

### Variation in C_L_ and C_D_ and the drag polar

Overall, *Draco* gliding lizards generated more lift than drag, C_L_ was always greater than C_D_. A steady AoA of 25.3 ± 2.8° with modulations in camber during the mid-glide phase resulted in the lizard using a C_L_ of 1.43 ± 0.22 (mean ± sd, n = 14) and C_D_ of 0.51 ± 0.11 (Fig. 3d,e). In the landing phase, C_L_ and C_D_ both increased at similar rates of 0.48 ± 0.17 m^−1^ and 0.52 ± 0.22 m^−1^, respectively. The average maximum C_L_ was 2.55 ± 0.40 and the average maximum C_D_ was 1.67 ± 0.56; both maxima occurred near the end of the landing phase just before touchdown. The average maximum lift-to-drag ratio was 3.22 ± 0.42 and peaked earlier during the mid-glide phase at 1.69 ± 0.73 m of horizontal travel (Fig. 3f).

**Figure 4:**
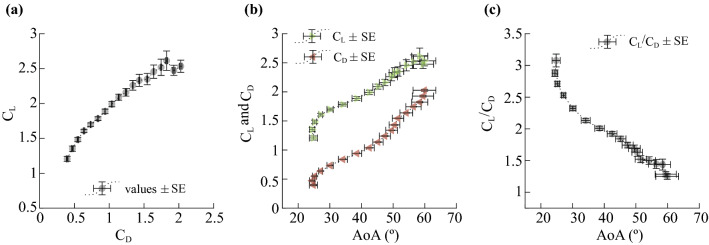
Aerodynamic performance of *Draco* gliding lizards recorded in the field motion capture arena. (**a**) A drag polar plot of the aerodynamic force coefficients; (**b**) their variation with the angle of attack (AoA). Note the expected gradual increase in each of the force coefficients with the increase in AoA up to a value of ~ 55°. (**c**) The lift-to-drag ratio shows a non-linear negative trend with respect to AoA. Because the underlying data are binned by *C*_*D*_, AoA (**b**,**c**) is not strictly increasing with respect to *C*_*D*_ or *C*_*L*_*/C*_*D*_ due to camber and inter-individual variation.

The drag polar plot derived from the kinematic and morphological data reveals a slightly concave downwards relationship between lift and drag, such that the highest lift-to-drag ratio occurred at low values of the observed coefficients (Fig. 4a). Variation in C_L_ and C_D_ with AoA show a well-resolved middle range from AoA of approximately 26°–50° where both coefficients increase steadily with increasing AoA (Fig. 4b). Coefficient values at *C*_*D*_ > 1.7 vary widely, with substantial differences in *C*_*L*_ between individual lizards increasing the variation present in the data set. Taken as a whole, these data show little change in *C*_*L*_ for *C*_*D*_ > 1.7 (Fig. 4a). At the high AoA extreme, values are represented by fewer data points, both within and among individuals, exhibit greater variability, and have no clear trend with AoA. Lizards typically used higher AoA values during the landing phase which was brief compared to the mid-glide phase (Fig. 4b). The low AoA extreme (< 25°) was mostly used in the transition from takeoff to mid-glide. The highest lift-to-drag ratio of ~ 3.1 was achieved at ~ 25° which corresponded to the steady AoA maintained by the lizard during the mid-glide phase (Fig. 4c).

### Effect of airfoil properties on C_L_, C_D_, and lift-to-drag ratio

We used the calculated average glide mechanics (see model fit section in “Materials and methods”) in three multivariate linear models to test the combined effect of AoA, camber, and the dihedral angle on C_L_, C_D_, and C_L_/C_D_ in the mid-glide phase. The model input was the difference between consecutive values of each averaged variable to ensure that the data were stationary with time and the residuals of the model output were not autocorrelated. The stationarity of the model input data was tested using the Augmented Dickey–Fuller test^29,30^. The test rejected the null hypothesis of non-stationarity with p < 0.01 for each variable. The residuals from the model output were tested for autocorrelation using the Durbin-Watson test^31^. The tests failed to reject the null hypothesis of the residuals being not autocorrelated with p > 0.45 for the C_L_, C_D_, and C_L_/C_D_ models. The landing phase was excluded from analysis due to persistent autocorrelation effects along with the non-linear effects of percentage camber associated with the steep drop in camber towards the end of the landing phase as the lizard bent its body dorsally and began to position itself for tree contact.

In the mid-glide phase, AoA and camber were significant and positive correlates of C_L_ and C_D_ but were negatively correlated with C_L_/C_D_. The equation of C_L_ was equal to 0.061(AoA) + 0.058(camber), F(2,23) = 8.12, p = 0.002 with R^2^ = 0.363. The equation of C_D_ was equal to 0.038(AoA) + 0.043(camber), F(2,23) = 12.81, p < 0.001 with R^2^ = 0.486. The equation of C_L_/C_D_ was equal to − 0.074(AoA) − 0.104(camber), F(2,23) = 8.03, p = 0.002 with R^2^ = 0.36. Dihedral angle was not a significant factor in any model and was not included in the final model estimations.

## Discussion

Our study provides real-world insight into the aerodynamic performance of *Draco* gliding lizards and how they actively modulate their orientation and airfoil properties to control aerodynamic force production. In our field motion capture arena, we found that wild-caught gliding lizards (*Draco* dussumieri) used an aerodynamic strategy that maintained their AoA at approximately 25° during mid-glide, sustaining a near-maximal lift-to-drag ratio while modulating camber to achieve different combinations of C_L_ and C_D_. In landing, the lizards rapidly increased their AoA to more than 55°, inducing a soft stall to maintain C_L_ while increasing C_D_ thus allowing them to minimize altitude loss while decelerating to land safely. Overall, these results supported our hypotheses of a drag polar with soft stall characteristics of low AR compliant airfoils, increases in camber during the glide associated with increasing force production, and control of AoA via mid-air changes in body posture.

### Aerodynamic performance of the compliant airfoil

Previous measurements of aerodynamic performance from live gliding animals have been limited to brief portions of the complete glide (but see^7^) or average performance values derived from the complete glide^2,3,18,32^. Both approaches lack the temporal and/or spatial resolution to quantify aerodynamic performance with respect to changes in body posture and airfoil configuration.

Here, we present a drag polar curve which encapsulates the interdependent aspects of the animal’s changing body shape and posture affecting the production of aerodynamic forces between AoA of 24° and 60°, the range of AoA used by the lizards in the motion capture arena. Our findings support the hypothesis that the lizard’s aerodynamic performance is similar to that of low AR airfoils at comparable Re and consistent with previous measurements of lift-to-drag ratio derived from the average glide angle of *Draco* lizards^32^. The lizard achieved its highest lift-to-drag ratio of ~ 3.1 at an AoA of ~ 25° followed by a steady increase in C_L_ and C_D_ up to 55°, and thereafter showing characteristic soft stall behavior with a rapid increase in C_D_ without drastic reduction in C_L_. Moreover, *Draco* gliding lizards produced higher lift-to-drag ratios at lower AoA compared to their gliding counterparts including gliding squirrels^18^, sugar gliders^3^, and gliding snakes^13^.

The lizards’ use of higher lift-to-drag ratios at lower AoA while sustaining efficient aerodynamic performance to upwards of 55° likely results from the combined influence of the airfoil camber, planform, and AR in gliding flight at Re ~ 10^4^. The primary aerodynamic force generating surface of the lizard is the open patagium (mean chord length = 5.6 cm) forming a Zimmerman shape^12,33^ like airfoil with AR of 1.41 ± 0.08 (mean ± sd, n = 14). Torres and Mueller (2004) showed that a rigid flat-plate Zimmerman airfoil of 1.5 AR at low Re (10^4^ to 10^5^) achieved higher maximum C_L_ at higher AoA compared to rectangular airfoils like those of mammalian gliders^12^. The maximum C_L_ (C_Lmax_) recorded here in *Draco* gliding lizards was 2.6 at around 58° AoA, compared to a C_Lmax_ of ~ 1 at 45° AoA for physical models of gliding squirrels^15^ (AR = 1) and a C_Lmax_ of ~ 2 at 35° AoA for model gliding snakes^13^. However, C_L_ calculated from animal experiments on gliding squirrels and sugar gliders show comparable aerodynamic performance to our derived drag polar curve^2,3,7,18^ albeit they were measured from only a brief portion of the complete glide^2,3,18^ (< 0.38 s) or with substantial measurement uncertainty^7^. Nonetheless, these results suggest that while the Zimmerman airfoil shape may be advantageous for *Draco* gliding lizards, animal gliders as a whole outperform static physical models, likely due to the effects of airfoil camber and flexibility. However, prior animal studies were unable to investigate these hypothesized effects due to experimental constraints.

In the lizard’s mid-glide phase, we observed a significant rise in C_L_ at low AoA associated with percentage camber varying between 8 and 10 and a dihedral angle of approximately 8°. Camber was significantly and positively correlated with C_L_ and C_D_. Bardera et al. (2020) showed higher flow velocities on the upper surface of cambered Zimmerman airfoils resulted in a region of low pressure and a mechanism for enhanced lift production^34^. Camber benefits potentially continued at high AoA with the drag polar curve showing soft stall characteristics past AoA of 55°. Pelletier and Mueller (2000) demonstrated that the flow on cambered surfaces remains attached over a larger range of AoA and over much of the airfoil resulting in delay of airflow separation with increasing AoA^35^. Furthermore, our results are corroborated by previous physical models of cambered airfoils at similar Re. Song et al. (2008) showed that for a rectangular latex wing membrane of 1.4 AR, camber resulted in a significant enhancement in lift at low AoA compared to rigid wings and exhibited a soft stall at AoA of over 40°^19^ (and see^36^). Similar benefits at low and especially high AoA regime have also been reported in studies of cambered Zimmerman airfoils^22,34^ and cambered rectangular plates at comparable Re^35,37^. Altogether, these inferences supported our hypothesis of higher airfoil camber leading to increased C_L_ and C_D_ during the glide.

Lastly, lower AR airfoils have the added advantage of wing tip vortices generating low-pressure regions over a significant portion of the airfoil. These flow structures can contribute to the overall lift production and energize the flow on the upper airfoil surface to keep the flow attached with increasing AoA, delaying stall^12,19^. Moreover, the delay in flow separation at high AoA could be facilitated by the placement of the lizard forelimbs at the leading edge of the patagium^21^, potentially acting as a leading edge slot and/or changing the leading-edge sweep angle^22,38^, although both these mechanisms remain untested.

### Aerodynamic gliding strategy

Theory indicates that gliding animals should operate at their maximum lift-to-drag ratio to maximize their glide range and minimize energy losses related to drag^39^. However, executing such a strategy demands that the animal operate in a narrow AoA window corresponding to their maximal lift-to-drag ratio, as shown on the drag polar curve. Furthermore, to control AoA, most gliding animals must continuously modulate their body posture in accordance to changes in their glide angle. However, the primary airfoil (patagium) anatomy restricts its rotation about the transverse axis and instead, couples it with changes in the animal’s body pitch orientation^20,23,25–27^.

Our results show strong evidence for such a strategy supporting our hypothesis that the lizard would vary its body pitch orientation to enable a high lift-to-drag ratio during mid-glide followed by high lift and drag forces at landing. The lizard steadily pitched upward throughout mid-glide, changing from an average pitch orientation of ~ − 24° to ~ 3° while closely matching its pitch rate (11.1 ± 2.7° m^−1^) with the mid-glide shallowing rate (11.0 ± 1.5° m^−1^). Thus, the average AoA was held at 25.3 ± 2.8° during this portion of the glide. Lift-to-drag ratio peaked for the lizards at about 25°, implying that the in-air change in body orientation during mid-glide serves to keep the lizard at a near-optimal configuration for maximizing glide distance. Aerodynamic measurements performed by Bahlman et al. (2013) on gliding squirrels hint at the presence of a similar gliding strategy of maintaining a mostly steady lift-to-drag ratio during the middle portion of a 18 m glide^7^. However, their study lacked a corresponding drag polar and AoA measurements needed to test the use of such a strategy.

Lizards were also found to pitch up sharply during the landing maneuver, reaching an average AoA of 40.4 ± 5.7°. According to the aerodynamic analysis this increases C_D_ by a factor of ~ 1.8 and increases C_L_ by a factor of ~ 1.3 compared to the ~ 25° mid-glide AoA. These high coefficients along with the shallowing glide trajectory correspond to large upward and rearward forces, removing kinetic energy from the lizard in part through drag losses and by conversion to potential energy and thus reducing the amount of remaining kinetic energy that would need to be absorbed by the body at landing.

### Limitations and future directions

In our aerodynamic performance measurements, we did not observe lizards gliding at AoA of less than 20° and have few brief samples from 20° to 24°, thus limiting our range of performance measurements between AoA of 24° and 60°. The absence of such glides could be an artifact of the experimental setup; the maximum takeoff height (observed between 4 and 5 m) was limited by the placement of the camera rig on the takeoff tree and the horizontal glide distance (5.50 m) was fixed based on the landing tree location. A longer glide distance might have elicited lower AoA. Furthermore, though our non-invasive experimental setup had the advantage of capturing real-world gliding behavior data, it limited the scope of data collection to kinematic and morphometric measurements of the animal. Therefore, we could not investigate specific mechanisms of aerodynamic control used by the animal. For example, it remains unclear if changes in airfoil camber are actively controlled by the animal’s body bending or are a passive outcome of the airfoil flexibility coupled with the changing aerodynamic load, or both. Also, in the case of *Draco* gliding lizards, the contribution of throat lappets as secondary force generating structures and in potentially delaying stall at high AoA^40,41^, remains to be explored. Lastly, although our analysis included the aerodynamic consequences of postural changes, it excluded investigations into the mechanisms that potentially produce these changes in the first place. Simulation studies have shown the role of tail in changing the pitch of *Draco* gliding lizards and gliding squirrels^14,15^ along with experiments on falling geckos showing the inertial effects of a rotating tail to control body roll and yaw^42,43^.

## Conclusion

Our study provides empirical measurements of the production and modulation of aerodynamic forces throughout complete glides in a wild population of flying lizards. We use these measurements to construct an aerodynamic performance landscape for the animal which highlights the critical role of the lizard’s planform, AR, and camber in generating substantial aerodynamic lift at AoA up to and over 55° without inducing stall. Furthermore, we show how the lizards precisely tune their aerodynamic performance via changes in body pitch that compensate for changes in glide speed and direction, keeping AoA close to 25° and resulting in a near-maximal lift-to-drag ratio throughout the mid-glide phase. In doing so, the lizard attains an optimal aerodynamic configuration for horizontal transport while allowing flexibility in the choice of C_L_ and C_D_ through changes in camber, enabling robust gliding characteristics to changing environmental conditions. Finally, our results provide insights into the physical mechanisms responsible for the unusual aerodynamic characteristics of actuated and compliant biological airfoils in gliding flight at Re ~ 10^4^ and lay the groundwork for future studies to generate more biologically relevant physical models of gliding animals.

## Materials and methods

### Field site and motion capture arena

The field site was an abandoned areca nut plantation located within the Agumbe Rainforest Research Station campus, Karnataka, India (13°31′04″ N, 75°05′18″ E) and previously described in Khandelwal and Hedrick, 2020^11^ (Fig. S2a). The field study was conducted from February to April 2017 during which 33 unique individuals (16 males and 17 females) inhabiting the plantation were identified (see SI-1). Glide data from these individuals were collected by constructing a motion capture arena on an approximately 6 m × 7 m patch of the plantation containing two areca nut trees 5.50 m apart with no trees in between (Fig. S2b–d). The two trees were designated as the takeoff and landing tree for glide recordings. An array of seven GoPro Hero4 Black cameras (GoPro, Inc) in wide field of view mode were used together to record the complete lizard glide from various viewing angles between the takeoff and landing tree (Fig. 1b, also see Figs. S3 and S4). Details of the motion capture arena and camera setup can be found in the supplementary material (see SI-2).

### Data collection

Glide data collection was performed from 9 March to 21 April 2017, during the *Draco dussumieri* mating season. Four data collection pauses of two days each were uniformly interspersed during the data collection period to reduce the effects of the team’s presence on lizard behavior at the field site. Glides were recorded between 9 am and 4 pm each day based on previous observations of lizard activity at the field site^11^. A complete glide recording began with capturing lizard(s) from the site and releasing them on the bottom of the takeoff tree in the motion capture arena. The number of lizards (male or female) on the takeoff tree varied from one to up to three during the day, often leading to intra-specific interactions on the tree. After one or more lizard(s) voluntarily climbed towards the top of the takeoff tree, all seven cameras were triggered to start video recording. Each recording duration ranged from a few minutes to up to ten minutes, depending on the time taken by a lizard to perform a voluntary glide. After completion of the glide, a scene calibration was performed, and the lizard was placed back on the takeoff tree. If the lizard had already performed three glides on the same day, its mass was measured on a digital balance (resolution ± 0.01 g) followed by photographing the lizard for morphometric measurements (Fig. S1). Post recording and measurements, the lizard was released back on the tree from which it was captured. No lizard was held captive for more than the duration of its recordings on a single day.

Appropriate permissions for conducting the study in the Agumbe Rainforest Research Station (ARRS) campus was obtained from the respective authorities. The study was approved by UNC Chapel Hill (UNC IACUC, protocol number 16-252.0) and ARRS authorities, and data collection was performed in accordance with the UNC Chapel Hill and ARRS guidelines.

### Data processing

3D position data ([x,y,z] coordinate) were obtained using the MATLAB (The MathWorks, Natick, MA, USA) package DLTdv^44^. For each glide recording, we manually digitized five body points on the dorsal side of the lizard in each frame of the complete glide (Fig. 2a). The five body points corresponded to locations on the anterior, middle, and posterior part of the lizard’s body, and the left- and right-wing tip. Each body point was digitized in all camera views in which it was visible (at least in two or more camera views per frame throughout the complete glide). These resulted in five tracks representing the complete glide trajectory with no missing digitization data. The mid-body point was used as a proxy for the center of mass and whole glide kinematic calculations including velocity and acceleration (Fig. S6). Thereafter, each glide was divided into the takeoff, mid-glide, and landing phase based on previously established criteria in Khandelwal and Hedrick, 2020^11^ (Movie S1). More information on calibration and data smoothing is available in the supplementary material (see SI-3).

### Data analysis

For each glide trajectory, we used the 3D position data of the five body points to calculate the roll, pitch, yaw, AoA, percentage camber, and the dihedral angle of the lizard at each instant of time for the complete glide (Fig. 2; also see Fig. S7a–f). Furthermore, the instantaneous acceleration along with the mass and the in-flight airfoil surface area of the individual were used to calculate the instantaneous values of C_L_ and C_D_ (also see Fig. S7g–i). However, the aerodynamic data analysis excluded the takeoff phase (t_takeoff_ = 0.43 ± 0.02 s, x_takeoff_ = 0.80 ± 0.08 m, mean ± sd, n = 14) and the glide trajectory just before touchdown (last 0.07 s of each glide, x = 5.32 ± 0.04 m) because the lizard patagium was typically seen to be opening or closing during those intervals. Nonetheless, our analysis covers ~ 82% of the complete glide of the lizard.

### Kinematic and aerodynamic variables

The 3D tracked five body points along with the velocity and acceleration values were used to calculate the following variables. The subscript (_x,y,z_) corresponds to the X, Y, or Z component of the measurement used in the calculation.

#### Yaw angle (^o^)

The angle made by the line joining the anterior and posterior point with the positive X axis at every instant of time (Fig. 2d).

$$yaw\; = \;{\tan^{ - 1}}\left( {\frac{{anterio{r_y}\;-\;posterio{r_y}}}{{anterio{r_x}\;-\;posterio{r_x}}}} \right)$$

where $anterior$ and $posterior$ refer to the tracked points on the lizard’s body.

#### Pitch angle (^*o*^)

Post yaw correction, the angle made by the line joining the anterior and posterior points on the lizard with the horizontal X–Y plane at each instant of time. A positive pitch angle raises the anterior point of the lizard and lowers the posterior point (Fig. 2e).

$$pitch = {tan}^{-1}\left(\frac{{anterior}_{z} - {posterior}_{z}}{{anterior}_{x} - {posterior}_{x}}\right)$$

#### Roll angle (^*o*^)

After correcting for pitch and yaw, the angle made by the line joining the left-wing tip and right-wing tip with the horizontal X–Y plane at each instant of time. A positive roll angle raises the left-wing tip and lowers the right-wing tip (Fig. 2f).

$$roll = {tan}^{-1}\left(\frac{{leftwing tip}_{z} - {rightwing tip}_{z}}{{leftwing tip}_{y} - {rightwing tip}_{y}}\right)$$

#### Percentage camber

After correcting for roll, pitch, and yaw, the ratio of the perpendicular distance of the mid-body point from the longitudinal axis to the distance between the anterior and posterior body point (chord length). The ratio is multiplied by 100 to give the percentage camber (Fig. 2h).

#### Dihedral angle (^o^)

After correcting for roll, pitch, and yaw, the average of the angle made by the line joining the mid-body point to the left- and right-wing tip with the horizontal X–Y plane. A positive dihedral angle raises the left- and right-wing tip above the horizontal X–Y plane (Fig. 2i).

#### Instantaneous glide angle ($\uptheta$, ^o^)

The angle defined by the inverse tangent of the ratio of the vertical component of velocity (${v}_{z}$) to the horizontal component of velocity (${v}_{x}$).

$$\theta = {tan}^{-1}\left(\frac{{v}_{z}}{{v}_{x}}\right)$$

#### Angle of attack (^o^)

The difference between the pitch angle and the instantaneous glide angle at each instant of time (Fig. 2g).

#### Airfoil area (m^2^)

A single video frame from the complete glide trajectory was used to calculate airfoil area. The frame corresponded to the lizard in glide when the patagium was completely stretched open. A total of 17 points were 3D tracked around the body periphery and included the head, lappets and the hindlimbs but excluded the tail. These 17 points were used to define an airfoil representing the total body surface area used to generate aerodynamic forces (Fig. 2c). The area was calculated by fitting a plane to the 17 tracked 3D points.

### Lift and drag calculations

Aerodynamic lift and drag were calculated using the angle ($\varphi$) between the drag vector, i.e. opposite to the velocity vector (${\varvec{V}}$), and the resultant acceleration vector (${\varvec{R}}$)^18^.

$${\varvec{R}}\text{ } = \, {\text{a}}_{\text{x}}\text{ + }{\text{a}}_{\text{y}}\text{ + (}{\text{a}}_{\text{z}}\text{ + 9.81)}$$

$$\varphi ={cos}^{-1}\left(\frac{-{\varvec{V}}.{\varvec{R}}}{|{\varvec{V}}||{\varvec{R}}|}\right)$$

The components of ${\varvec{R}}$ perpendicular and opposite to the lizard’s direction of travel were multiplied by the lizard’s mass ($m$) to give the absolute lift and drag force, respectively.

$$L\; = \;\left| R \right|\sin \varphi \cdot \;m;\,\,D\; = \;\left| R \right|\cos \varphi \cdot m$$

A visualization of the resultant aerodynamic force and its components of lift and drag are shown in Movie S1.

Instantaneous coefficients of lift and drag (i.e., *C*_*L*_ and *C*_*D*_) were calculated from the kinematic and airfoil data using the following equations:

$${C}_{L} = L{\left(\frac{1}{2}\rho S{v}^{2}\right)}^{-1}$$

$${C}_{D} = D{\left(\frac{1}{2}\rho S{v}^{2}\right)}^{-1}$$

where $\rho$ is air density (1.225 kg m^−3^), $S$ is the airfoil area, $v$ is instantaneous airspeed, and $L$ and $D$ are the absolute lift and drag forces, respectively. Note that $S$ is assumed to be constant at the measured fully open value, so these instantaneous calculations will not produce accurate results for the beginning and end of the glide when the lizard patagium is being opened or retracted.

#### A note on airfoil area for aerodynamic calculations

We share an observation emphasizing the importance and dependence of the airfoil area on the calculation of coefficients of lift and drag in our study. This is of course a simple mathematical statement, but it raises practical problems when applied to gliding animals that have relatively small wings but may use much of their body surface in gliding. During initial analyses of our dataset, we used only the area of the patagium, measured by hand using the morphometric data images, as the airfoil area. This resulted in implausibly high values for the coefficients, leading us to shift to using the planform area of the entire lizard, including head, body, patagium and proximal portions of the limbs for aerodynamic force coefficient calculations, approximately doubling the area compared to the patagium alone. Comparison between this dataset and other studies of animal gliding should keep this methodological distinction of airfoil area in mind, it strongly influences the value of *C*_*L*_ and *C*_*D*_ but not their ratio since in that case the area terms cancel. Furthermore, this result emphasizes the significant contribution of the mostly flat body parts of the glider, apart from the primary wing surface, towards generating aerodynamic forces as exemplified in the extreme by gliding snakes which lack any morphologically distinct wing.

### Drag polar curve

The set of corresponding values for C_L_, C_D_, and AoA were determined as follows from their instantaneous values using a sliding window approach applied to C_D_ from 0.35 to 2.05 in increments of 0.1, a range ensuring that each window included data from at least 5 individuals (Fig. S8). For each window mid-point, we identified all instantaneous C_D_ values from the mid-point ± the increment of 0.1. Within this data subset we calculated the average C_D_, C_L_ and AoA for each individual lizard represented. From this set of individual means we calculated the inter-individual mean, inter-individual standard deviation, and standard error at each of the C_D_ window mid-points (Fig. 4). Note that the values contributing to each mid-point overlap by 50%, for example, the mid-points at 0.35 includes the range from 0.25 to 0.45 while the mid-point at 0.45 includes the range from 0.35 to 0.55. The C_D_ overlap was included to lessen the effect of the artificial window size on the resulting glide polar curve.

### Model fit of aerodynamic force coefficients with airfoil properties

We divided the fixed horizontal glide distance into intervals of 0.10 m starting from 0.80 to 5.32 m along the + X axis and identified all values of AoA, percentage camber, dihedral angle, C_L_, C_D_, and lift-to-drag ratio (C_L_/C_D_) within each interval. The start and end distances corresponded to the average horizontal distance covered based on the criteria established for aerodynamic data analysis. For each variable, we calculated the mean and standard deviation of all its values within each interval, allowing us to maximize the variation captured for each variable. The raw data used are shown in Fig. S7. Thereafter, the difference of consecutive values of each averaged variable were used to investigate the effects of airfoil properties (AoA, camber, and dihedral angle) on the lizard’s aerodynamic force production capabilities (C_L_, C_D_, and C_L_/C_D_) using a multi-variate linear regression model – [C_L_ or C_D_ or C_L_/C_D_] ~ AoA + camber + dihedral.

### Average glide mechanics

We used a similar approach as described in the model fit analysis above to calculate the average glide mechanics with glide progression over the fixed 5.50 m glide distance. In each 0.10 m interval between 0.80 and 5.32 m along the + X axis, we identified all values of each kinematic and aerodynamic variable. However, instead of averaging all values of each variable in each 0.10 m interval, we first averaged the intra-individual values of each variable. Subsequently, we calculated the inter-individual mean and inter-individual standard deviation of each variable in each 0.10 m interval, the results of which are shown in Fig. 3.

### Analysis and statistical methods

All calculations, linear regression model fits (‘fitlm’ function), and statistical analysis was performed in MATLAB version r2021a. The average metrics reported follow the format of (mean ± sd) and are calculated from a sample size of 14 corresponding to 7 males and 7 females, unless otherwise mentioned. List of other MATLAB functions used include ‘adftest’ function for the Augmented Dickey Fuller test, ‘dwtest’ function for the Durbin Watson test, ‘ttest2’ function for the two-sample t-test.

## Supplementary Information

Supplementary Video 1.

Supplementary Information 1.

## Supplementary Information

The online version contains supplementary material available at 10.1038/s41598-022-05739-1. The study dataset and analysis code can be accessed at 10.6084/m9.figshare.16602368.
