# Supplementary material for: Combined effects of body posture and three-dimensional wing shape enable efficient gliding in flying lizards
Source: Sci Rep. 2022 Feb 2;12:1793. doi: 10.1038/s41598-022-05739-1 (PMC8811005; doi:10.1038/s41598-022-05739-1)
Supplement: Supplementary file 2 — Supplementary Information 1. [file 41598_2022_5739_MOESM2_ESM.pdf]

Supplementary Information for

Combined effects of body posture and three-dimensional wing shape enable efficient gliding in flying lizards

Authors: Pranav C. Khandelwal[a*] and Tyson L. Hedrick[b]

[a]Max Planck Institute for Intelligent Systems, 70569 Stuttgart, Germany
[b]Department of Biology, University of North Carolina at Chapel Hill, Chapel Hill 27599, USA

*Pranav C. Khandelwal
Email: pranav@is.mpg.de

Supplementary Information Text

SI-1. Tree and *Draco* tagging

Prior to data collection, from 24 February until 8 March 2017, an extensive tree and *Draco dussumieri* survey was carried out at the field site to uniquely identify individuals and their spatial distribution. All trees in the site were marked with a unique letter and number combination. The letters were the rows (short side of the site), and the numbers were the columns (long side of the site), together forming a grid like pattern. The markings were made with dark red ink on the tree and placed approximately 40 cm from the ground to reduce any influence that the markings might have on the behavior of animals in the vicinity (Figure S2.a inset). A total of 912 trees were marked spanning the entire length and width of the site resulting in a tree density of approximately 13 trees per 100 m^2.

Along with tree marking, *Draco*(s) spotted in the plantation were captured using a contraption. The contraption was made of a 6 m aluminum pole with a horseshoe shaped wire attached to a pliable tong at one end which was wrapped with a thick, soft padding and a cloth hanging from it. The horseshoe shaped wire could be wrapped around the tree trunk above the *Draco* and motivated the lizard to climb down on the tree or glide to a nearby tree. Once within reach, the lizard was caught by hand, and the tree location on which it was captured was recorded. The captured *Draco* was marked by painting a unique number using white correction ink (Kores Eraz-ex Aqua Fast Dry Correction Fluid) on the dorsal side (Figure S1.a). This was followed by measuring the mass of the lizard on an electronic balance (Figure S1.c) and placing the lizard on a graph paper (grid size of 1 cm) and capturing images for morphometric measurements. Multiple images of the lizard were taken from the ventral and dorsal sides with the limbs, body, and tail almost flat on the graph paper and the camera optical axis positioned perpendicular to the graph paper. An image was also taken with the patagium spread for wing area calculations (Figure S1.b). The sex of the lizard was identified based on their dewlap length with the female lizards having a significantly shorter dewlap compared to the males[1]. The lizard was then released on the same tree from which it was captured.

During the course of the entire field season, there were instances when certain *Draco* had almost lost their markings over time or due to shedding of their skin. On such occasions, the lizard was recaptured and given the same unique number as before. The field study resulted in a total of 33 marked individuals consisting of 16 males and 17 females.

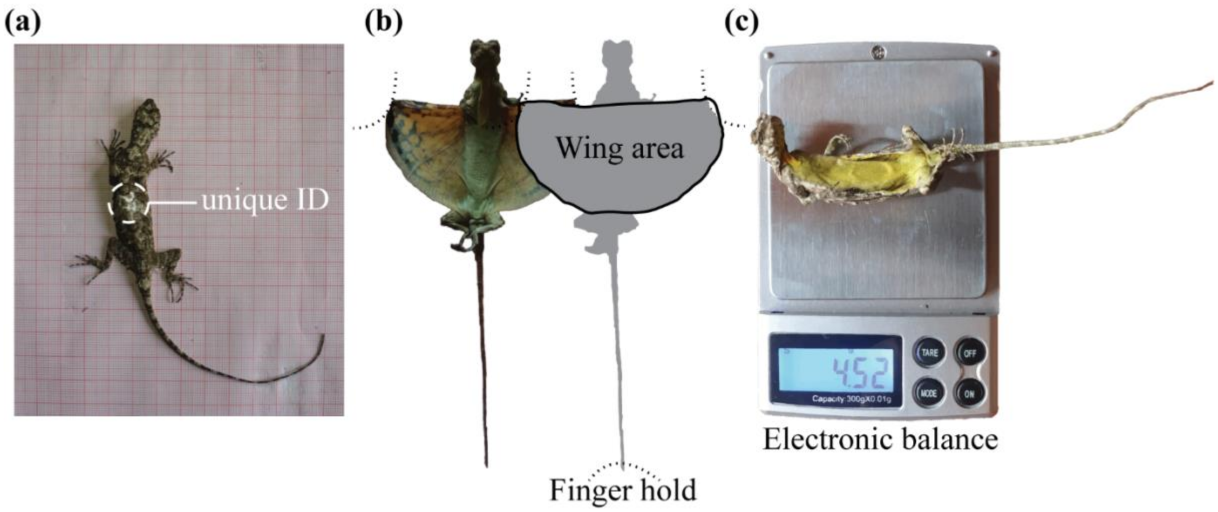

Figure S1. *Morphometric measurements of the lizard.* (a) Each lizard was marked with a unique identification number on the dorsal side, shown by the white dashed circle. Images were taken by placing the lizard flat on a graph paper (grid size of 1 cm) for morphometric measurements. (b) Images were also taken of the lizard on the graph paper by laying it on its dorsal side and extending the patagium open (as freely permitted by the lizard) to calculate the patagium surface area. (c) The lizard was placed on an electronic balance to measure its mass in grams with a resolution of ± 0.01 gm.

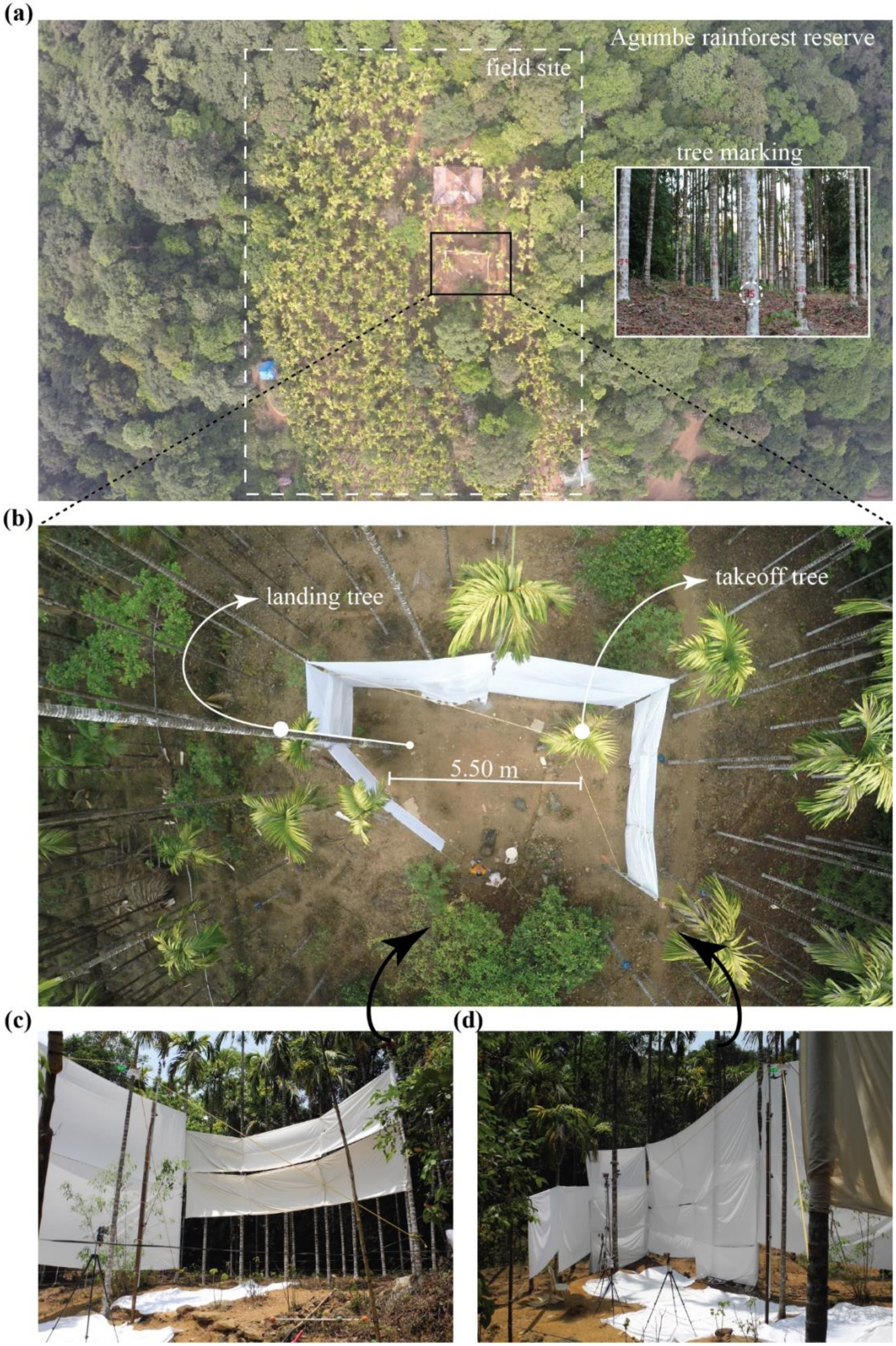
(a)
Agumbe rainforest reserve
field site
tree marking
(b)
landing tree
takeoff tree
5.50 m
(c)
(d)

Figure S2. *Field site and the motion capture arena*. (a) Overhead view of the abandoned *Areca catechu* plantation (field site) surrounded by the Agumbe rainforest reserve. The plantation was ~115 m x ~60 m in size. The inset shows the unique tree markings (shown by the white dashed circle) painted to identify each tree and its location. The black rectangle shows the location of the motion capture arena in the plantation. (b) Overhead view of the motion capture arena consisting of the takeoff and the landing tree 5.50 m apart and cordoned off from the remaining plantation using white cotton fabric sheets. (c) and (d) are two views of the motion capture arena taken from the positions of the corresponding arrowhead. They show the white sheets spanning the height of the surrounding trees along with sheets placed on the ground to provide a high contrast background for 3D tracking.

SI-2. Motion capture arena

The patch of land on which the motion capture arena was constructed was selected to represent the average glide distance of approximately 5 m observed in our previous study (Khandelwal and Hedrick, 2020) of natural gliding behavior at this field site (Figure S2.a)[2]. The patch was enclosed by a mix of shrubs and rainforest on the south-east and areca nut trees on all other sides. The only two trees within the patch were designated as the takeoff and landing tree for glide recordings. The arena was constructed by cordoning off the two trees from the rest of the site using white cotton fabric sheets on all sides except the south-east. The white sheets spanned almost the entire tree height and were also spread on the ground between the takeoff and the landing tree (Figure S2.b; also see Figure S2.c-d). The sheets encouraged the lizard to glide towards the designated landing tree by eliminating other tree options and provided a high contrast background for video recording and 3D motion tracking.

We used an array of seven GoPro Hero4 Black cameras (GoPro, Inc) in wide field of view (FOV) mode (horizontal FOV of 118.2°), which together recorded the complete glide of the lizard between the takeoff and landing tree. The seven cameras were categorized into three groups based on their position between the takeoff and the landing tree. The camera mounted on the takeoff tree and on the pole adjacent to the takeoff tree formed the takeoff camera group. They were used to record close-up recordings of the takeoff phase. Three cameras on the ground recorded the glide between the takeoff and landing tree and formed the glide camera group. The camera mounted on the landing tree along with the one placed adjacent to it formed the landing camera group. They were used for close-up recordings of the landing phase. Reconstructing 3D kinematics required that the animal be seen simultaneously by at least two cameras.

Takeoff camera group. The group consisted of two cameras. One was mounted close to the top of the takeoff tree looking downwards towards the ground and capturing close-up top view recordings of the takeoff phase. The second camera was mounted on a pole adjacent to the takeoff tree and at a similar height as the first camera. The second camera was tilted, pointing towards the white sheets on the tree to capture the side view of the takeoff phase. Both cameras recorded video at 240 frames per second (fps) and 480p resolution (cameras 1 and 2 in Figure S3.a and Figure S4; also see Figure S3.c).

Glide camera group. The group consisted of three cameras placed on the ground in a staggered height and position configuration between the takeoff and landing tree. The first camera was placed closer to takeoff tree looking upwards with part of the sky as the backdrop. The FOV captured the complete glide. The second camera was placed approximately midway between the takeoff and landing tree and had the white sheets as the backdrop. It also captured the complete glide. The last camera was placed closer to the landing tree and captured part of the mid-glide phase and the complete landing phase. All three cameras recorded at 120 fps and 1080p resolution (cameras 3, 4, and 5 in Figure S3.a and Figure S4; also see Figure S3.b).

Landing camera group. The group consisted of two cameras. The first camera was mounted on the landing tree looking downwards towards the ground. This allowed close-up recordings of the top view of the landing maneuver. The second camera was placed adjacent to the landing tree on the ground, pointing towards the white sheets on the tree and provided a side view of the landing phase. Both cameras recorded at 120 fps and 1080p resolution (cameras 6 and 7 in Figure S3.a and Figure S4; also see Figure S3.d).

Each of the seven cameras was accompanied by an 80 mm cooling fan, a walkie-talkie (Motorola Talkabout Radio MH230R), and a 16000 mAh external battery pack (Anker Astro E5 – Model A1208) (Figure S3.b). The cooling fan was mounted approximately 8 cm away from the back of the camera using a wire frame to damp vibrations during recording. The airflow was towards the back of the camera and prevented it from overheating during extended recording. The external battery pack charged the camera and powered the cooling fan, allowing for the cameras to remain on and record during the entire day. The walkie-talkie was used to receive audio notes and beeps that were used for temporal alignment of the video frames from all cameras for 3D reconstruction. All seven cameras were connected to a GoPro Wi-Fi remote to start and stop recording.

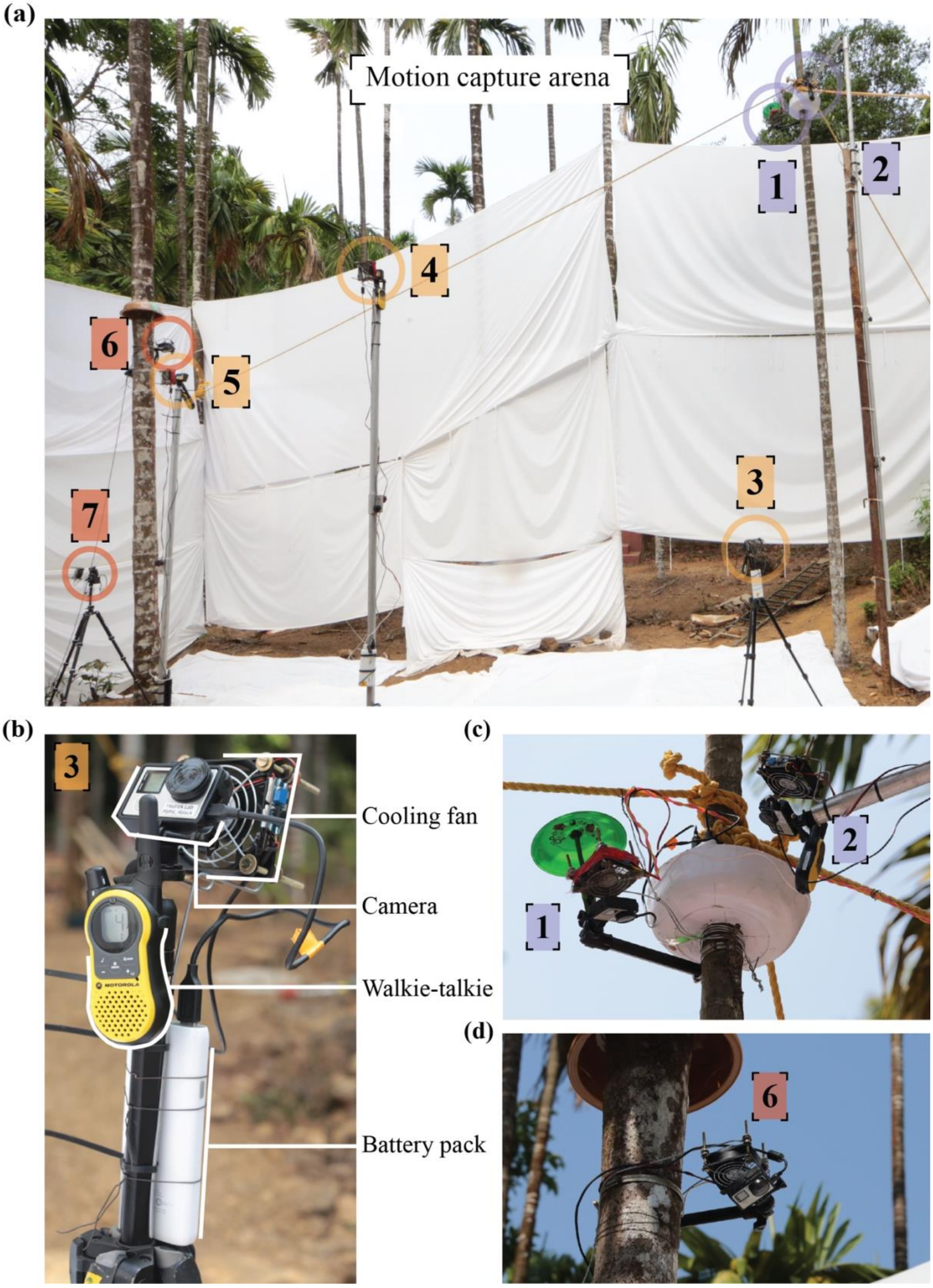
(a)
Motion capture arena
1
2
3
4
5
6
7
(b)
3
Cooling fan
Camera
Walkie-talkie
Battery pack
(c)
1
2
(d)
6

Figure S3. *Camera rig and recording setup in the motion capture arena*. (a) Side view photograph of the motion capture arena in the field with all seven cameras marked. The tree adjacent to camera 1 is the takeoff tree and the tree adjacent to camera 7 is the landing tree. Cameras 1 and 2 formed the takeoff group, cameras 3, 4 and 5 formed the glide group, and cameras 6 and 7 formed the landing group. (b) Close-up view of camera 3 in the motion capture arena. Each camera was equipped with a cooling fan and battery pack to prevent overheating and enable day long recording. The walkie-talkie was used for temporal alignment of video frames and taking audio field notes. (c) Close-up view of the takeoff cameras 1 and 2. Camera 1 was mounted on the takeoff tree and camera 2 was on a L shape pole erected next to the takeoff tree. A cooling fan was mounted at the back of both cameras and a single walkie-talkie was used for both. (d) Close-up view of camera 7 mounted on the landing tree. The fan at the back of the camera was equipped with an external power switch which allowed us to turn off the fan as soon as the lizard landed to prevent any injury to it.

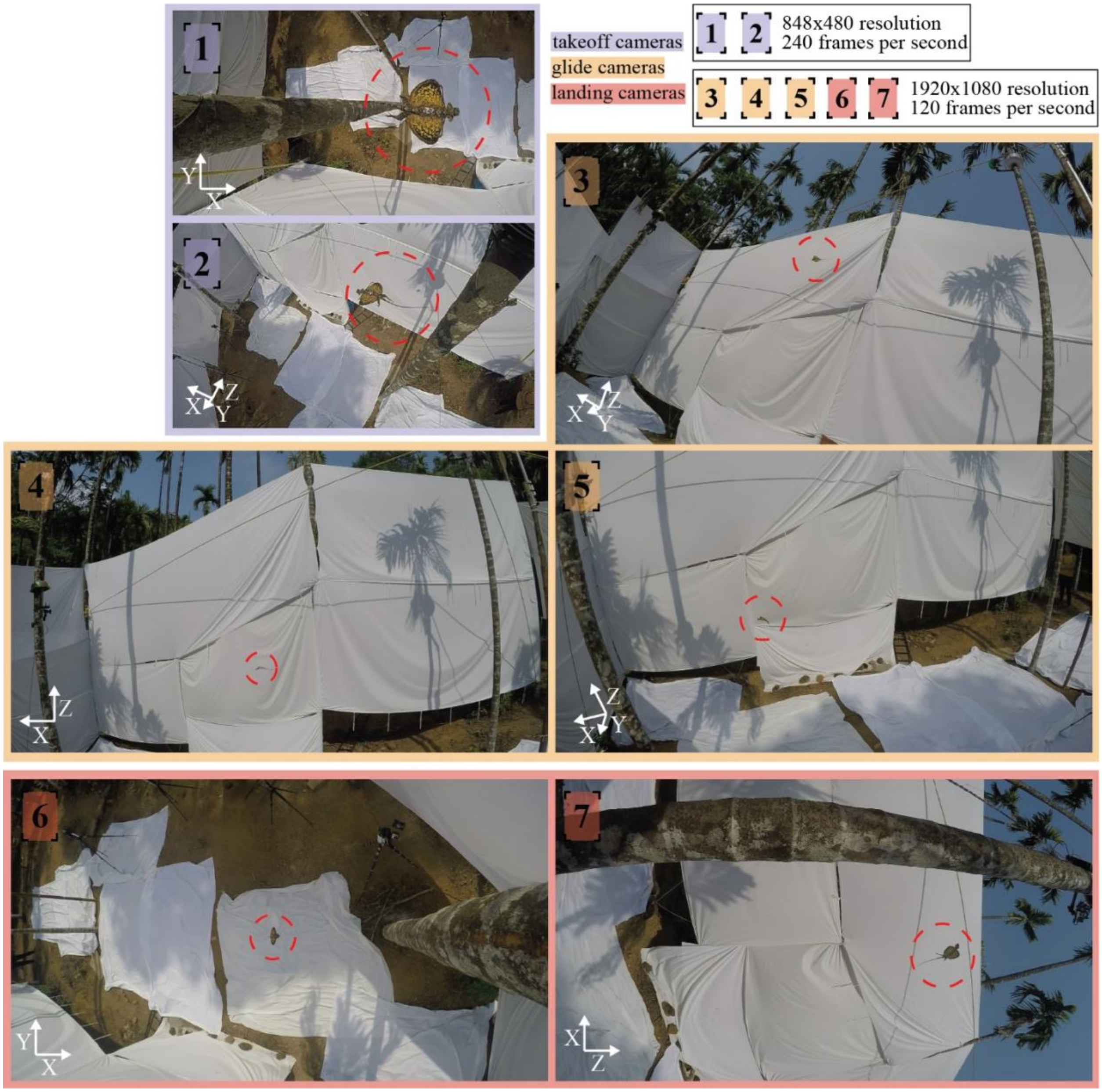

Figure S4. *Views from all seven cameras in the motion capture arena*. Snapshots from each camera view. The axes in each camera view show the approximate orientation of the camera. The positive X axis is along a straight line joining the takeoff and the landing tree, the positive Y axis is pointing away from the white fabric sheets, and the positive Z axis is pointing upwards opposite to the direction of gravity. The seven snapshots show different time points during which the lizard (highlighted using a red dashed circle) is visible in each camera view during the complete glide.

SI-3. Video data collection

On each data collection day, wild flying lizards were captured from the field site using the contraption and the tree on which each lizard was captured was noted. Based on the abundance and ease of capture of the lizard, at times, up to three lizards were captured in quick succession within the field site. The captured lizards were carried to the motion capture arena in a transparent, well-ventilated plastic box. The lizard(s) were released at the bottom of the takeoff tree in the motion capture arena. If the *Draco* captured for recording was unmarked, the lizard was marked after completing all the glide recordings for that day to ensure that the fresh marking did not affect the gliding behavior in the arena. Once one or more lizards climbed towards the top of the takeoff tree, all seven GoPro cameras were triggered to start recording using the GoPro Wi-Fi remote controller.

At the start of the recording, 10 audio beeps were broadcasted on the walkie-talkies which would be used for temporal alignment of video frames across all cameras for 3D reconstruction. Each recording varied between a few minutes to up to 10 minutes depending on the time taken by the lizard to perform a voluntary glide. No external perturbation was provided to the lizard to induce a glide. However, when there were multiple lizards present on the takeoff tree, the intra-specific interactions could have motivated the lizard to glide towards the landing tree. Once the glide was performed, audio notes were taken to note the unique ID of the lizard that performed the glide (also verifiable from camera 1 FOV), the time of the day, and the air temperature. The cooling fan on the landing tree camera was turned off to ensure no harm to the lizard followed by performing a scene calibration using a wand and a tennis ball. The wand was moved in the entirety of the motion capture arena in different orientations and varying its height above the ground. After the wand movement, a tennis ball was tossed in the vertical direction which would be used for gravity alignment of the scene. Finally, another 10 audio beeps were broadcasted to all walkie-talkies before ending the recording for all cameras.

Post completion of the recording, the lizard was caught from the landing tree and placed back on the bottom of the takeoff tree. If three complete glides of an individual had been recorded, the mass of the lizard was measured, and images of the lizard laid flat on the graph paper were taken for morphometric measurements before being released on the same tree from which it was caught. During the field season, there were instances when the lizard escaped from the motion capture arena after gliding to the landing tree. In such cases, we were unable to carry out mass and morphometric measurements of the lizard and instead, used the measurements from the closest day on which the same individual was captured and measured.

SI-3.1. Scene calibration

The cameras were calibrated for 3D reconstruction using a structure-from-motion approach with bundle adjustment based on the end points of a wand of known length that was moved through the calibration volume. The wand was constructed using a pipe of 1.8 cm diameter and 1.04 m in length. Three distinct colored balls were placed on the wand. Orange ball on one end and red on the other end. A green ball was placed 26 cm from the red ball. The red and orange ball helped easily identify the two ends of the wand and the green ball was used to distinguish between the two ends for consistent digitization for scene calibration. Calibration calculations were performed using the easyWand MATLAB package[3]. The scene was aligned to gravity by tracking the motion of a tennis ball tossed in the air and aligning to its acceleration. This also provided an independent measure of calibration quality by comparing the measured acceleration magnitude to expected gravitational acceleration; values range from 97.63 to 100.32 % with a median of 99.05 % of the gravitational acceleration (9.81 ms^{-2}).

SI-3.2. Video analysis

Once calibration was complete, five body landmarks were tracked through the complete trajectory. The anterior point was identified as the place where the head of the lizard joined the body. The posterior point was identified as the place where the tail joined the body. The left- and right-wing tips were the two extremities of the wing, clearly demarcated as the two end points of the *Draco* along the transverse axis. The mid-body point was roughly the mid-point between the anterior and posterior body point and corresponded to a distinct pattern on the dorsal side of the lizard, often overlapping with the marking of the unique ID.

Each track was smoothed using a smoothing quintic spline weighted by the 95% confidence intervals of the 3D reconstruction uncertainty. There were no missing points or gaps in the digitized tracks used for data analysis. Each glide was rotated and translated to place the takeoff tree on the origin and

the landing tree on the positive X axis. The raw position data and the smoothed data are shown in figure S5. The first and second derivatives of the smoothed and rotated tracks provided the instantaneous 3D velocity and acceleration values for the complete glide. The complete XZ glide profile along with individual velocity and acceleration values for all 24 glides are shown in Figure S6. The velocity and acceleration values were used to divide the complete glide into the takeoff, mid-glide, and landing phase as described previously in Khandelwal and Hedrick (2020)[2]. A visualization of a sample complete glide track along with the glide phases and the five tracked body points are shown in Movie S1. A basic summary of metrics for each glide phase is reported in Table S1.

Table S1. Duration, horizontal distance, and average glide angle for each glide phase across all 14 individuals. The values reported are mean ± sd.

	**Takeoff**	**Mid-glide**	**Landing**
**Duration (s)**	0.43 ± 0.02	0.72 ± 0.08	0.55 ± 0.10
**Horizontal distance (m)**	0.80 ± 0.08	2.63 ± 0.29	2.09 ± 0.30
**Glide angle$_{avg}$ (°)**	-35.9 ± 3.2	-36.2 ± 2.6	-12.8 ± 3.7

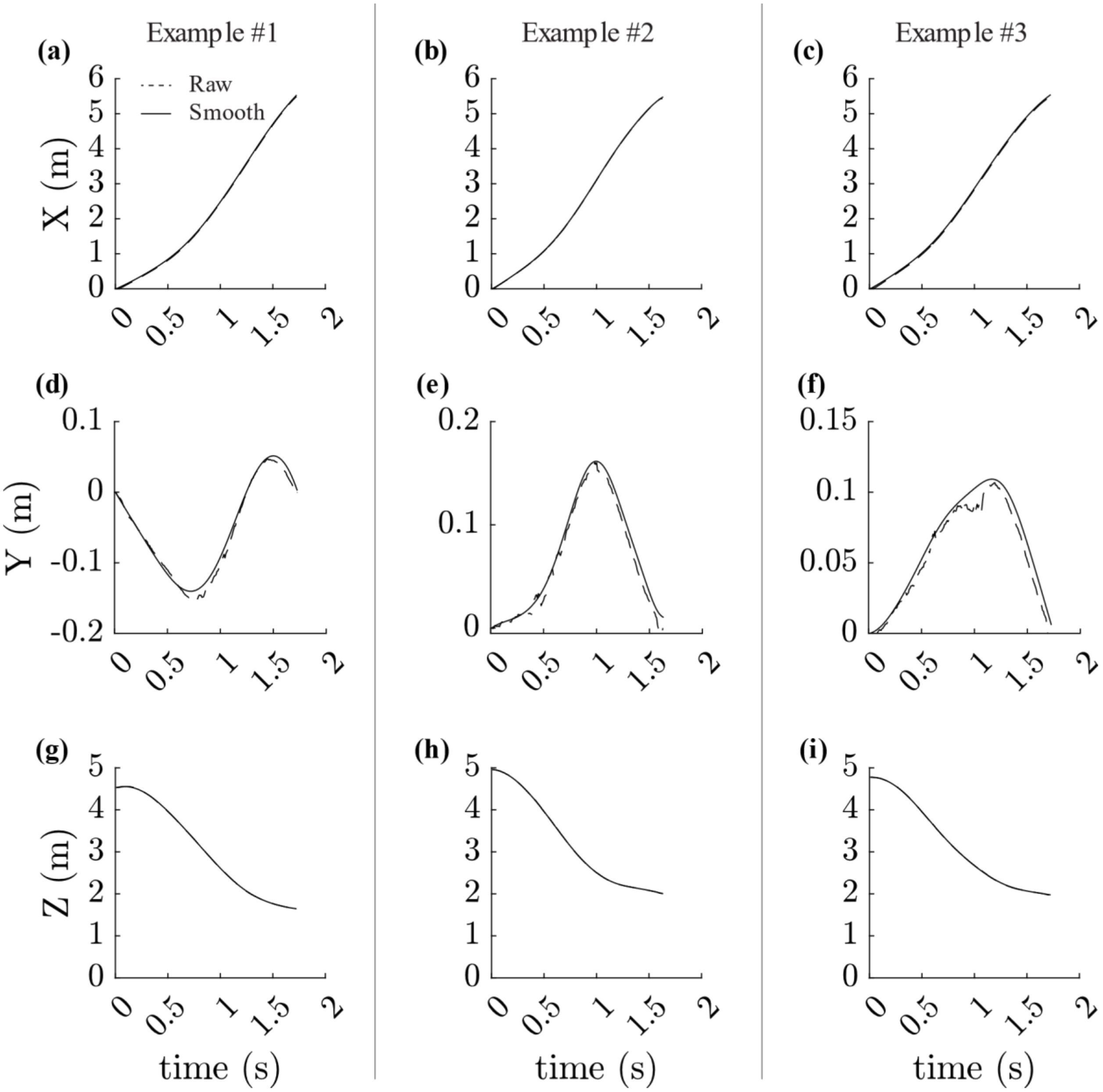

Figure S5. *The raw and smoothed XYZ position data for three sample glides.* The raw position data were exported using the DLTdv MATLAB package[4] and the DLT calibration coefficients[3]. Thereafter, each track was smoothed using a smoothing quintic spline weighted by the 95% confidence intervals of the 3D reconstruction uncertainty estimated by DLTdv.

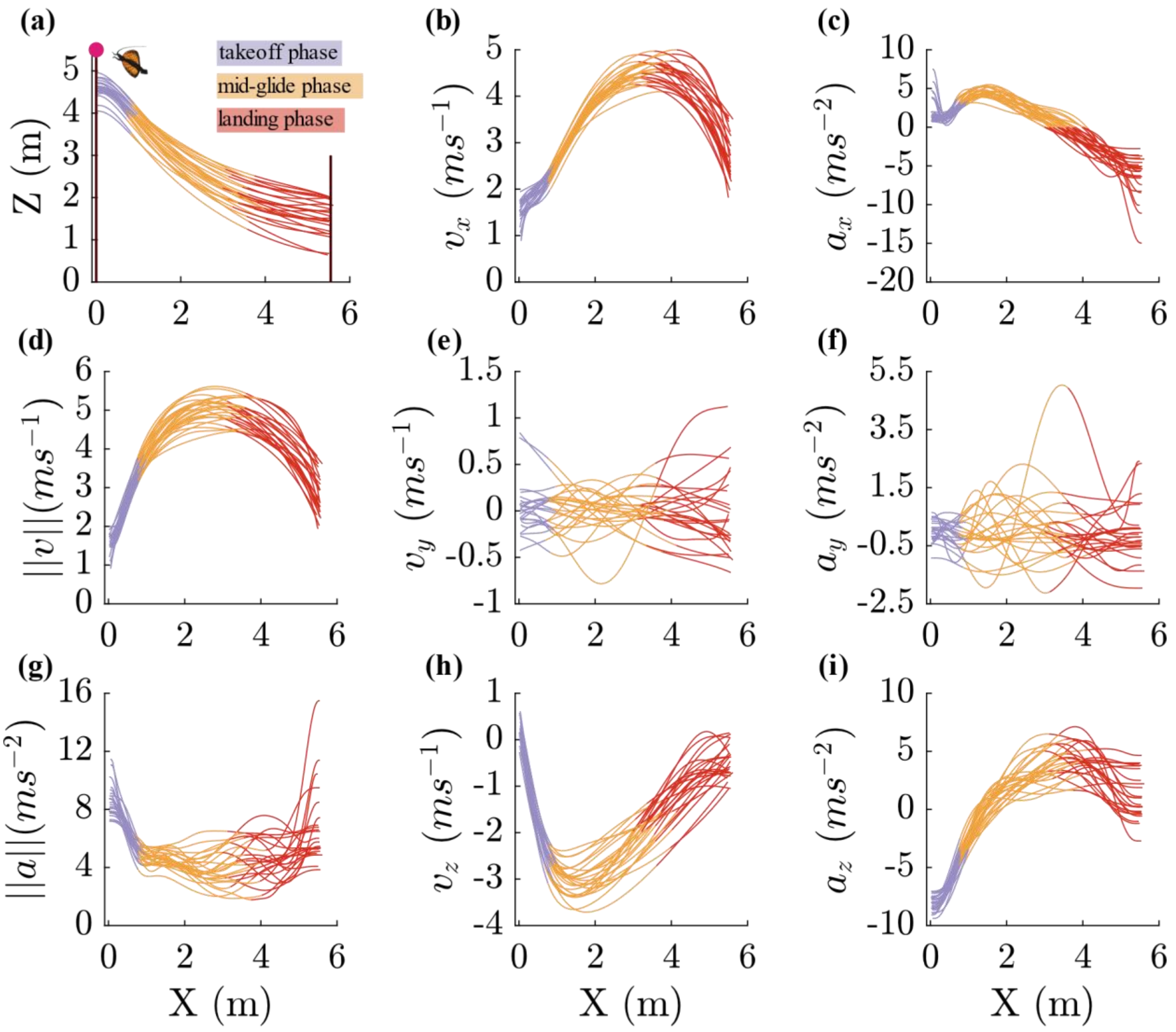

Figure S6. *Kinematics of all 24 glides.* Panels (a), (d), and (g) show the side view profile of all glides recorded along with the magnitude of speed and acceleration. The glide trajectory is divided into takeoff, mid-glide, and landing phases based on the definitions described in Khandelwal and Hedrick (2020)[2]. Panels (b), (e), and (h) show the x, y, and z components of the velocity vector during the glide. Panels (c), (f), and (i) show the x, y, and z components of the acceleration vector during the glide. Note the start value of a_z close to 9.81 ms^{-2} indicating the ballistic dive phase of the takeoff phase.

SI-4. Calculation of rate for pitch, AoA, C_L, C_D, and the shallowing rate

The rate of each variable was calculated by fitting a separate linear regression model of the variable with the horizontal distance (X) for all 24 glides. The slope from each model was used as the rate value for the corresponding glide. All 24 rate values were used to calculate the intra-individual average followed by the inter-individual average which is reported in the main manuscript. To calculate the shallowing rate, we fit a model of the instantaneous glide angle with X.

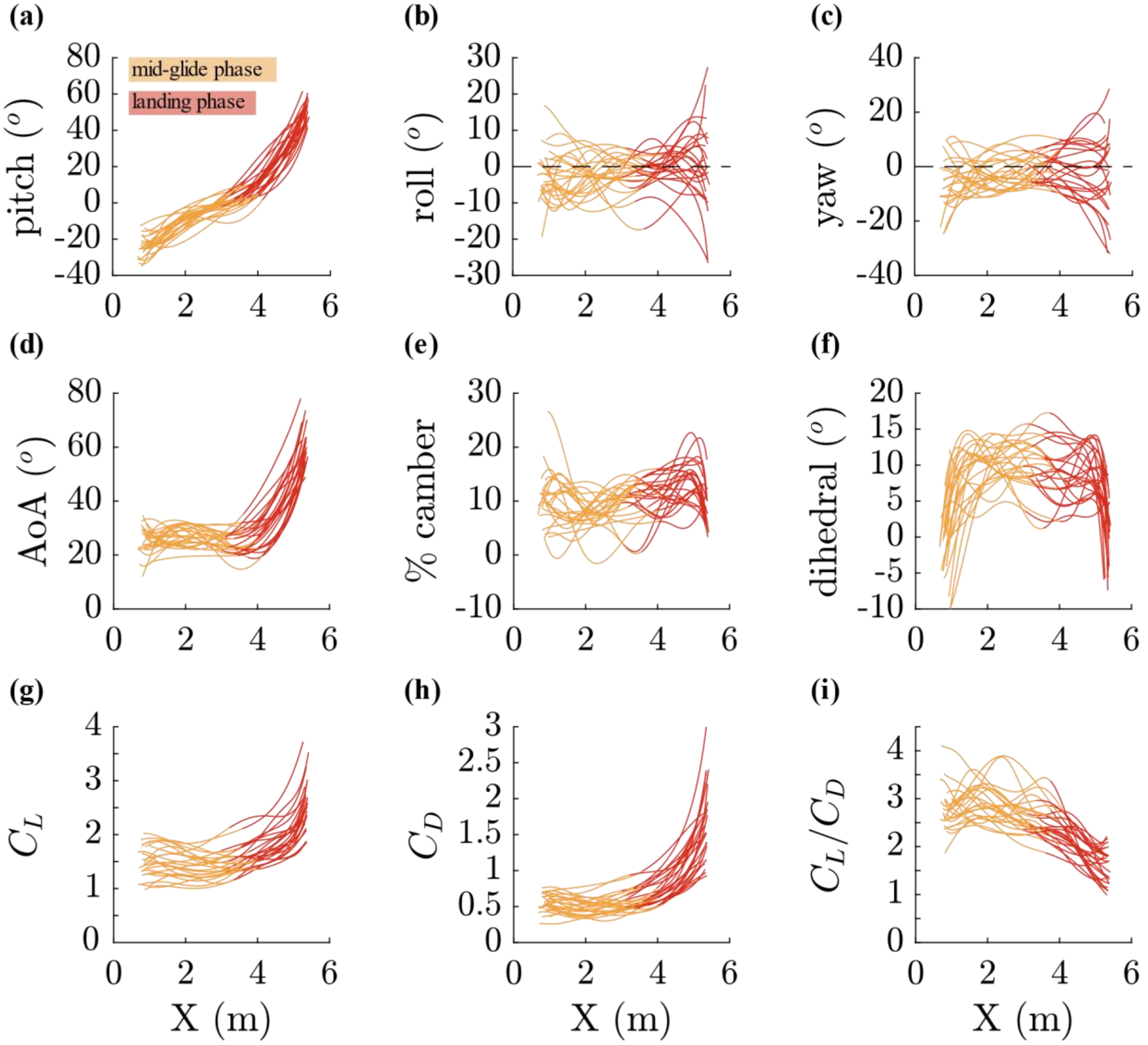

Figure S7. *Aerodynamic properties of all 24 glides restricted to the duration of complete wing deployment.* (a) The pitch angle showed steady increase during the mid-glide phase followed by a more rapid increase in the landing phase. (b) and (c) show the highly variable roll and yaw during the glide with an average value of close to 0°. (d*)* *Draco* gliding lizards maintained a mostly constant AoA during the mid-glide phase followed by rapid increase in the landing phase. (e) Percentage camber varied throughout the glide with a drastic drop towards the end as the lizard prepared for tree contact. The average camber during the mid-glide phase was significantly lower than that in the landing phase. (f) Dihedral angle was held mostly steady during the complete glide with similar values in the mid-glide and the landing phase. (g) and (h) show the coefficient of lift and drag values during the glide. C_L was larger than C_D with C_D increasing more rapidly than C_L in the landing phase. (i) The lift-to-drag ratio varied slightly during the mid-glide phase followed by a rapid decrease in the landing phase.

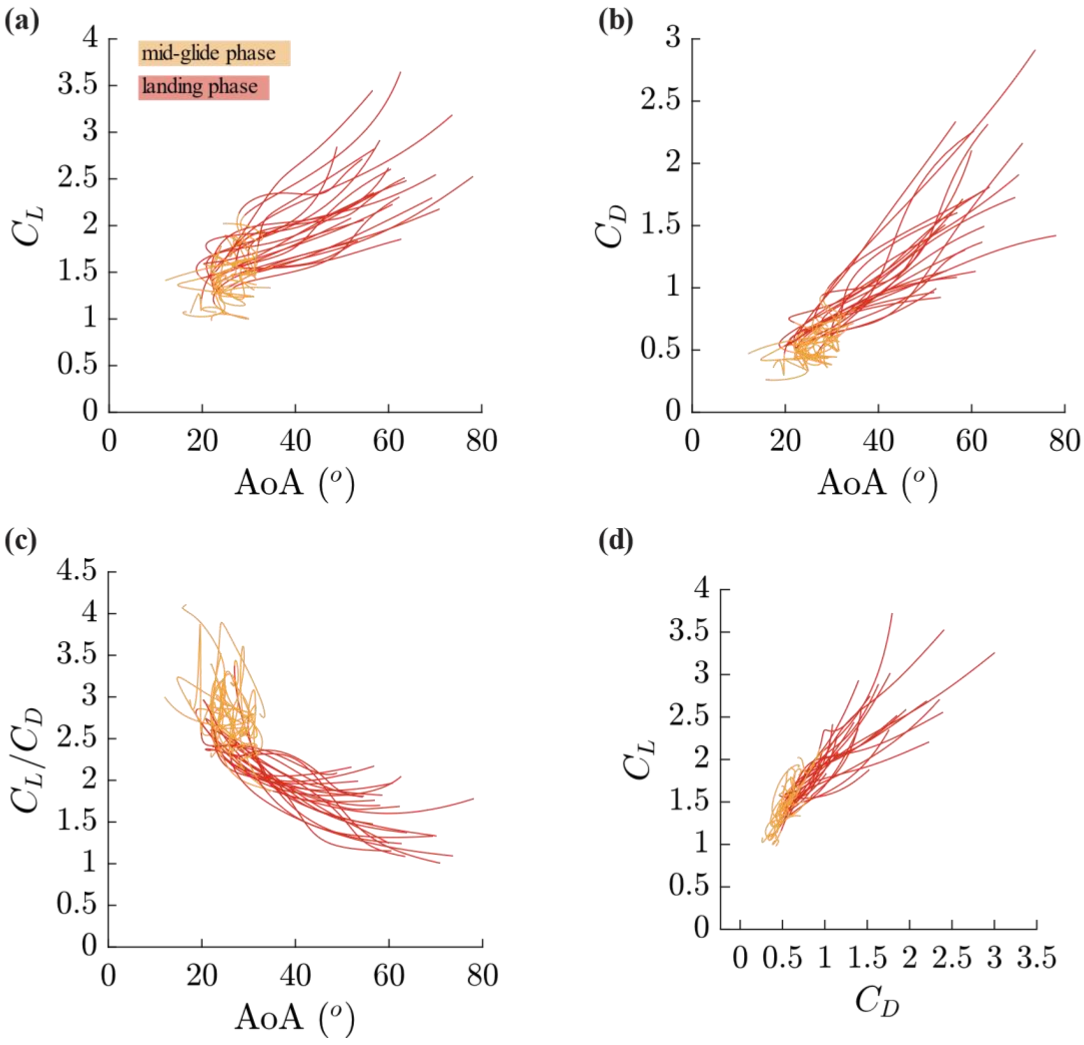

Figure S8. *Individual aerodynamic data of all 24 glides used to construct the drag polar curve for the Draco gliding lizard.* The data shown is restricted to the range in which the wing was completely stretched open. The high degree of variation along with the reduced number of data points can be seen at AoA > 55° leading to larger uncertainty in the average drag polar curve as shown in the main manuscript (Figure 4.b and 4.c).

Legend S1 Movie

3D animation of a sample glide and the change in body posture and airfoil properties during the glide. The left animation shows the complete glide with the red solid circle representing the anterior tracked point of the lizard. The trajectory color denotes the speed of the lizard during the glide. The takeoff, mid-glide, and landing phase are denoted by the purple, orange, and red color under the trajectory, respectively. The right animation is a zoomed in view of the five tracked body points corresponding to the [x, y, z] position in the left animation. It shows changes in the body roll, pitch, yaw, and the airfoil camber and dihedral during the complete glide. The resultant aerodynamic force and its components of lift and drag are represented by the three arrows emanating from the tracked mid-body point of the lizard.

Legend S1 Dataset

The dataset and analysis code are available in the figshare repository and can be accessed at -
DOI: 10.6084/m9.figshare.16602368

The dataset includes kinematic and morphometric measurements of 24 3D tracked glides in the field motion capture arena. The 24 glides are recorded from a total of 7 male and 7 female lizards. Using these measurements, we calculate the airfoil properties and the aerodynamic force coefficients. The dataset is available as a MATLAB formatted data file labeled ‘fs17_data_v4.mat’. A detailed description of the contents of the dataset are described in the ‘readme_v4.txt’ file. The analysis and the figures for the main manuscript can be produced using the MATLAB script ‘fs17_aerodynamics_v3_upload.m’.

SI References

1. Sreekar, R. *et al.* Photographic Capture-Recapture Sampling for Assessing Populations of the Indian Gliding Lizard Draco dussumieri. *PLoS One* **8**, e55935 (2013).

2. Khandelwal, P. C. & Hedrick, T. L. How biomechanics, path planning and sensing enable gliding flight in a natural environment. *Proc. R. Soc. B Biol. Sci.* **287**, 20192888 (2020).

3. Theriault, D. H. *et al.* A protocol and calibration method for accurate multi-camera field videography. *J. Exp. Biol.* **217**, 1843–1848 (2014).

4. Hedrick, T. L. Software techniques for two- and three-dimensional kinematic measurements of biological and biomimetic systems. *Bioinspiration and Biomimetics* **3**, 034001 (2008).
